# Death and diminishment: parasitoid flies (Diptera: Conopidae) reduce foraging efficiency before killing their bumblebee host

**DOI:** 10.1007/s00442-025-05679-3

**Published:** 2025-02-25

**Authors:** T’ai H. Roulston, Anne Larsen, Amber D. Slatosky

**Affiliations:** 1 Department of Environmental Sciences, University of Virginia, Charlottesville, VA 22908 USA; 2 Mountain Vista Governor’s School, Middletown, VA 22645 USA; 3 Ecology and Evolutionary Biology, Princeton University, Princeton, NJ 08544-2016 USA; 4 Ophthalmology Department, University of California-Irvine, Irvine, CA 92617 USA

**Keywords:** Parasitism, Sublethal effects, Conopidae, Bombus, Behavioral immunity

## Abstract

Host–parasitoid interactions typically result in either a dead parasitoid or a dead host. Understanding the effects of parasitoid success on a host can be estimated primarily as how much an early death curtails host reproduction. When parasitoids attack the nonreproductive caste of social insects, however, the effects are not the reduced reproduction of the host but rather the sum reduction in host contributions to its colony. In addition to the loss of host workdays due to premature death, there is potential for additional cost through reduction in foraging efficiency as the infection develops. To better understand these pre-lethal effects, we allowed conopid parasitoid flies (Conopidae) to infect workers from a colony of the bumblebee *Bombus impatiens* (Apidae) in the lab and then moved the colony to an outdoor location. Bumblebee foragers were monitored using RFID technology and an automated analytical balance positioned between the colony and the outside environment. We found that infected bumblebees foraged similarly to uninfected workers halfway through their fatal infections. Starting at day 6–7, however, infected bees took fewer trips per day, which resulted in a significant reduction in resources returned to the colony over the last 3 days of the experiment. Both infected and uninfected bees were likely to remain out of the colony at night after their fourth day foraging, but infected bees started staying out sooner. These pre-lethal effects of a developing parasitoid add to the negative effects of a shortened lifespan on host contribution to its colony.

## Introduction

Parasites vary in their effects on hosts, from mild illness to sterility to death (Abbate et al. 2015; Cory 2017). Since the detrimental effects of parasites on hosts can range widely, selection on hosts to resist parasites should also range widely, such that hosts do not invest in costly defenses that outstrip the damage they are likely to receive. These trade-offs in host–parasite interactions have led to a rich literature on parasite virulence (Day et al. 2007; Leggett et al. 2013) and the concomitant responses of their hosts (Hosken 2001; Schwenke et al. 2016), showing complex and often geographically varied patterns of coevolution (Anderson and May 1982; Thompson 1994; Gandon and Michalakis 2002; Lajeunesse and Forbes 2002; Laine 2006).

Parasitoids are a particular type of parasitic organism whose success typically ends with host death (Jervis and Ferns 2011). Their offspring gain entry into the host by various means and feed from host tissues until the host dies. With such a dire tradeoff between success of the parasitoid and death of the host, the evolutionary possibilities should be substantially truncated, hinging primarily on two factors: successful acquisition of the host through behavioral strategies, and successful development of the parasitoid in the presence of the host’s physiological or behavioral immune defenses (De Roode and Lefèvre 2012). Despite these binary outcomes, selection on the evolution of host defense has various potential nuances (Kraaijeveld and Godfray 1997; Fellowes and Godfray 2000; Schmid-Hempel 2022). If defense is costly and parasitoids are rare, low investment in defense should be selected in most circumstances (Straub et al. 2015). In addition, even if parasitoids are common, death by parasitoid may not be particularly costly to the host if other mortality risks are similar, such that lifespan is not greatly reduced, or if a reduction in lifespan does not substantially reduce reproduction. This could happen if a host was post-reproductive or, in the case of social insects, if the host is a nonreproductive member of a social group (Straub et al. 2015).

In insects such as ants, termites, and the social bee and wasp species, reproductive individuals spend most or all of their lives within nests, surrounded by nonreproductives that guard the nest and forage for food and nest materials (Bourke 2007). Since the fitness of nonreproductives arises through the success of their colony (Gardner and West 2014), their behaviors should favor colony survival and growth more than their own survival. In fact, it is common for the nonreproductive castes of social insects to self-sacrifice in defense of the colony from predators or disease (Shorter and Rueppell 2012; Cremer et al. 2018). An analogous form of sacrifice would be reduced self-preservation behaviors or reduced immune investment if the associated costs to colony functions (e.g., reduced foraging efficiency) were greater than the expected gains through greater longevity. Foraging is an activity with high potential for exposure to many sources of mortality, including parasitoids, and thus foragers typically live much shorter lives than the primary egg laying caste (Heinze and Schrempf 2008). If parasitoids can kill hosts without greatly reducing their expected lifespan or their foraging efficiency, then parasitoids may not greatly reduce their hosts’ contributions to their colony. In this case, hosts would not be under strong selection pressure to alter their behavior or develop immune responses to defend themselves against this single source of risk, especially if there are costs to defense. Estimating the effects of parasitoids on host contributions to colonies, however, is challenging in natural environments.

One of the few well-studied systems involving social insects and their susceptibility to parasitoids is bumblebees (Apidae: *Bombus* spp.) and conopid flies (Conopidae). Bumblebees are social insects in which queens remain in their nest after production of their first brood of workers. Workers tend the brood, defend the nest and forage for resources in the environment. Conopids are a family of obligate parasitoids that utilize other insects for hosts, especially wasps and bees (Smith and Peterson 1987). They are especially abundant in bumblebees, with several conopid genera utilizing different species of bumblebees on several continents and typically killing their hosts 10–12 days after infection (Schmid-Hempel and Schmid-Hempel 1996; Abdalla et al. 2014; Malfi and Roulston 2014). Bumblebee infection rates have frequently been found to be over 30% of foragers in the field, with rates as high as 80% recorded (Gillespie 2010; Davis et al. 2015). The effects of conopids on bumblebee colony reproduction, however, have never been directly measured, though estimates have been made based on simulating their effects through worker removals from active colonies (Müller and Schmid-Hempel 1992) and estimating their effects using a field parameterized model of bumblebee colony reproduction (Malfi et al. 2018).

Understanding the costs of parasitoids on bumblebee colony reproduction requires not only knowing the cost of an early death but also any pre-lethal effects that the developing infection may impose. Infected bees appear to forage up until the day of their death, even though most of their abdominal cavity is taken up by the developing fly. Thus, any effects of the fly that reduce the host’s foraging efficiency should also be considered as impacts on potential colony reproduction. Currently, the only pre-lethal effects known are a tendency for infected bumblebees to (1) remain out of the colony at night, potentially as a behavioral immune response (Müller and Schmid-Hempel 1993); (2) bring back pollen to the colony less frequently (Schmid-Hempel and Schmid-Hempel 1991), and (3) bring back different kinds of pollen to the colony (Schmid-Hempel and Stauffer 1998).

Here, we describe an experiment that examines how resource collection efficiency of infected bumblebees changes over the course of a conopid infection. We do this by allowing bumblebees to forage in an open environment following controlled lab infections by conopids and monitoring the resources returned to the colony through the use of RFID technology and the continual recording of individual bee weights as they go out to forage and return with resources.

## Methods

### Study site

All field work was carried out at Blandy Experimental Farm (Virginia, United States of America, 39.06° N 78.06° W), an ecological field station of the University of Virginia. The 290 hectare property is composed of young fields with herbaceous perennials, isolated forest fragments, and a 72 hectare public garden.

### Study organisms

This study was carried out in early to mid-July, 2021 using a single commercial colony of the common eastern bumblebee, *Bombus impatiens* Cresson 1863, purchased from Koppert Biological Systems (Howell, Michigan). Upon their arrival, the bumblebee colony was placed in a large (15 m × 11 m), parasitoid-free screenhouse with flowering plants so that workers could gain foraging experience. All bees that foraged in the screenhouse over a 7-day period were captured and marked individually using RFID tags (Microsensys, GmbH, Erfurt, Germany (Microsensys, GmbH, Erfurt, Germany, type: mic3^®^-TAG 16 k, System: iID^®^-2000-G, ISO15693, size: 1.6 × 1.9 × 0.5 mm^3^, Carrier Frequency: 13.56 MHz) and numbered tags (Betterbee^®^ queen tags) following cold-induced sedation. They were then returned to the colony and the colony placed in the lab for several days prior to exposure to parasitoids. The colony contained ~ 100 workers at deployment. Conopid flies were captured from the wild at Blandy Experimental Farm in Boyce, Virginia, a mountain meadow near Capon Bridge, West Virginia and a warm season grass meadow near Sperryville, Virginia. All flies were brought to the lab on ice and placed into a foraging arena the same day. Despite the prevalence of conopid infections in bumblebees in the region (Malfi and Roulston 2014), female conopids can be difficult to find. In total, 9 female conopids were caught in ca. 15 person hours of searching. All but one was of the species *Physocephala tibialis* (Say, 1829) with one individual of *P. marginata* (Say, 1823).

### Lab infection protocol

**Fig. 1:**
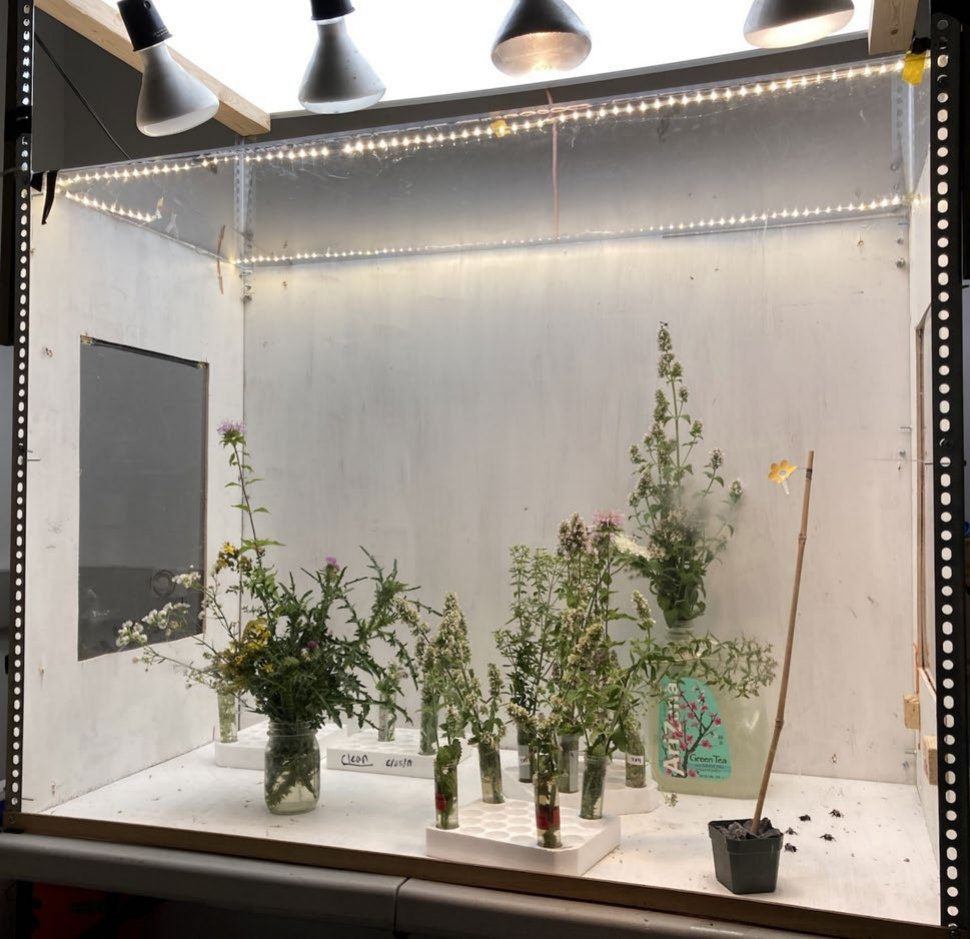
Foraging arena. Five bumblebees and 4–5 female conopid flies were in the arena at once. Conopids typically perched on plants surveying the arena. After warming up, bumblebees flew in the arena visiting flowers

Conopids were placed together into a 1.3 m × 1 m × 0.7 m foraging arena stocked with cut flowers from fields adjacent the field station. The chamber was well lit with a 4000 lm LED overhead panel (Lithonia Lighting model CPANL 2 × 4), a string of 120 LED lights (1200 lm total) and 4 additional 120 V flood lights (Fig. 1). Flowers served as feeding resources both for bees and conopid flies. Multiple female conopids (4–5 at a time) were placed together because preliminary trials in 2020 revealed both a lack of aggression between female conopids housed in a common environment and great variation in their individual propensity to attack bees in the artificial arena. Keeping them together thus maximized the efficiency of getting multiple bumblebees attacked in a short period of time.

Sixty bumblebee foragers were randomly assigned to a conopid exposure treatment, either placed in a foraging environment with conopid flies or being separated from the colony and held in vials for a similar amount of time. During exposure trials, bumblebees were placed into the conopid attack arena in groups of five and monitored for attacks. Since we were unable to determine which attacks were successful, we allowed individuals to get attacked up to four times. In some cases, an individual conopid attacked the same bee in short succession as if its first attempt had been unsuccessful. No attempt was made to distinguish individual conopids, but we tracked the total number of attacks experienced by each bee. Each group of five bees was left in the foraging arena for approximately 1 h, when attack activity had greatly diminished. At that point, the first group was removed (even if some individuals had not been attacked) and another group was placed into the arena. All exposed and unexposed bees were returned to the colony after the attack trials. A total of six trials were carried out over 2 days (i.e., 30 bees were in the lab exposure group and 30 in the lab unexposed group).

### Tracking foraging efficiency in the field

The bumblebee colony was moved to the field on July 12, 2021, the 4th day after the first groups of bees were exposed to conopids in the lab and the 3rd day after the last groups of bees were exposed. The delay in deployment was designed to allow the conopid eggs to hatch, estimated at 2 days (Schmid-Hempel and Schmid-Hempel 1996), and allow the larvae to begin consuming the host. Keeping the bees in the lab during the initial days, rather than deploying them longer, reduced the likelihood of bees dying in the field of other causes prior to the conopids reaching a later developmental stage. Since we could not recognize a conopid infection prior to examining the bumblebee after it had died, we had to successfully retrieve bees at the end of the foraging period to assess their parasitoid status during the experiment.

**Fig. 2:**
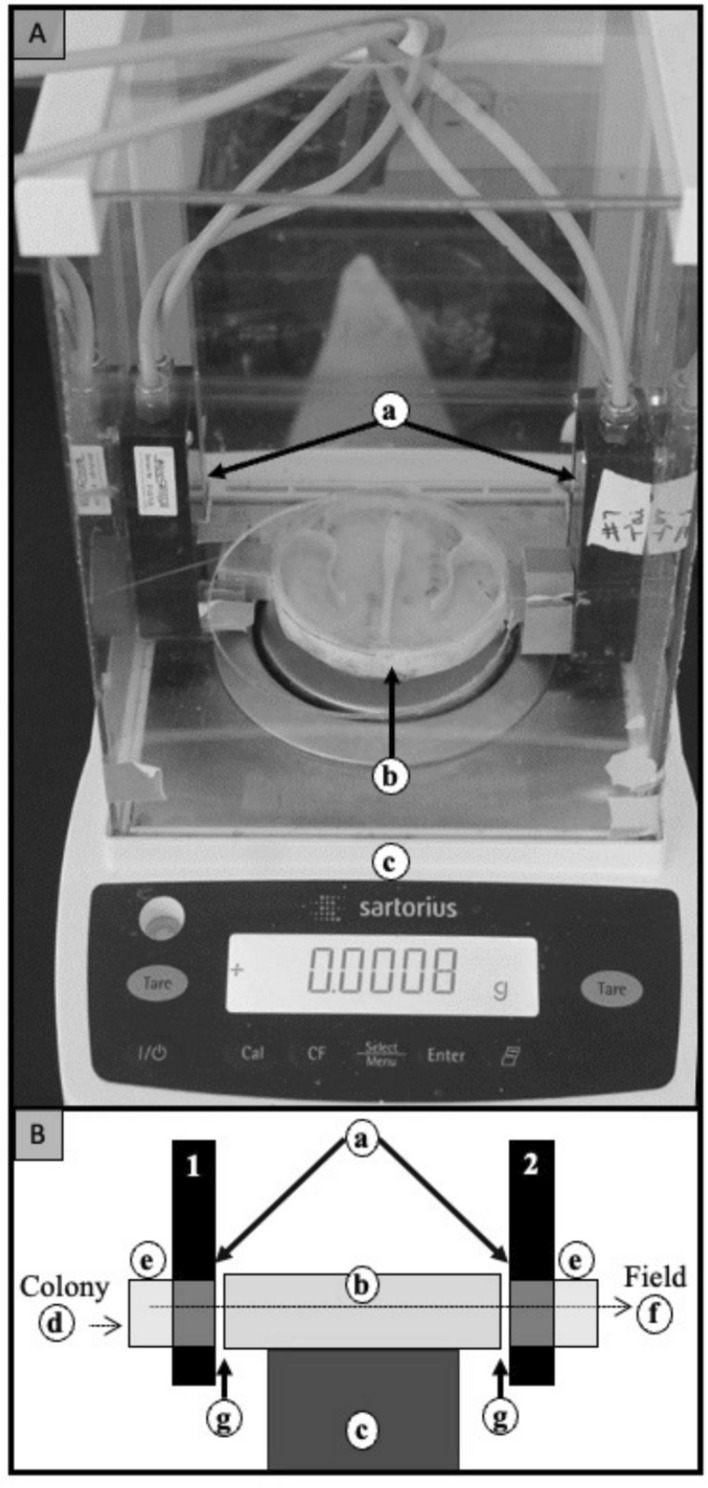
Image (**A**) and schematic (**B**) of analytical balance showing pathway of bees from the colony to the field. Bees leave colony (d) via exit tube (e), pass through one RFID reader (a), navigate a maze (b) resting on the analytical balance, pass through a second RFID reader (a), enter a second exit tube (e), then depart into the outside environment (f). The maze allowed the balance to stabilize while the bee was on it. Small gaps (g) were necessary to ensure free movement of the scale surface. A motion-activated camera took short video clips of each bee on the maze, and the balance fed time-stamped weight data continuously to a computer

The colony was placed in a wooden shed in a hedgerow beside an early successional field. Upon exiting the hive box, the bees were forced to pass through an RFID reader onto an analytical balance (Sartorius Entris 64-1S), out through a second RFID reader and then outside to forage. On the surface of the analytical balance was a covered maze that slowed the passage of the bees so that the weight on the scale could stabilize (Fig. 2). The maze was 3-D printed (Creality Ender 3, Shenzhen Creality 3D Technology Co., Ltd, Shenzhen, China) and covered with transparent film (0.18 cm thick Hygloss^™^ Acetate Sheets—Hygloss Products, Inc., Wallington, NJ). It slowed bees enough for the scale to stabilize but not so much that the bees became confused and failed to cross. Scale readings were continuously fed to a computer (~ 5 readings per second) and stored with a timestamp. Following the experiment, all scale readings were read into a computer program written in R (R Development Core Team 2021) that would detect weight shifts consistent with a bee entering or exiting the scale and determine whether the scale had stabilized based on the standard deviation of 3 consecutive scale weights between stepping on and stepping off behavior. This yielded a file of useable time-stamped scale shifts. To assess a bee foraging trip, a stable weight had to be recorded for both leaving and entering the colony. Timestamps on the RFID data were matched to the scale data to identify each bee. In addition, a motion-activated camera (Logitech C910 webcam) was placed over the scale and recorded time-stamped videos each time a bee passed. The use of the camera often allowed resolution of the weight of an individual bee even if two bees were on the scale at the same time. It also usually showed the bee’s physical tag to further confirm its identity. A bee’s weight was estimated as the stable weight of the bee on the scale minus the stable weight of the scale before the bee got on the scale (this allowed for weight estimates if a second bee was sitting on the scale already). Since bees do not always carry the same amount of food in their crop when they exit the colony, we always estimated the resources returned during a foraging trip as the weight of the bee when it returned minus the weight when it left.

After 5 days of foraging, the colony was provided with pollen and an artificial nectar supply and the colony door was set so that bees could return but not exit. This was done in late afternoon because bumblebees with several days foraging experience commonly exit the colony late in the day and spend the night in the field, returning the next day. Thus, there is never a time in which all bees are naturally in the colony. Although some bees did return through the ‘in-only’ door, many were deterred by the change in the doorway and had to be manually captured and returned to the colony. Bees that could not be manually captured immediately typically returned to the field and had to be pursued at the colony entrance later that day or the next day.

All experimental bees that were recaptured at the end of the foraging time were separated from their colony and placed in an empty bumblebee colony box in the lab with 50% sugar water ad libitum. For the next 2 weeks, this box was checked daily and dead bees were removed and examined repeatedly for the presence of a large conopid by prying open the rear abdominal opening. At the time that a conopid kills a bumblebee, it typically occupies nearly the entire abdominal cavity and positions itself such that its paired spiracles are against the abdominal opening, making it easy to recognize advanced conopid infections. We did not do complete dissections to examine for small conopid larvae, as our interest was in the effects of later stage conopid infections at the time of foraging. Based on the number of days post-foraging that the bee died, we could distinguish between conopid infections originating from our exposure treatment and those originating in the field. Bees that died in the lab within 12 days of their exposure to conopids in the lab and contained a mature conopid larva were considered to have been infected during lab exposure. Bees that died in the lab with mature conopid larvae in their abdomen 14–20 days after the lab exposure period (including bees that were not exposed to conopids in the lab) were considered to have been infected in the field during foraging. In those cases, conopids would have been in an early stage of development while the bee was foraging, given a 2-day latency period for conopid eggs and a total foraging duration of 5 days. One bee exposed in the lab but not observed to be attacked died with a mature conopid larva in its abdomen 10 days after its lab exposure. This was inferred to have been an unobserved lab attack because the development time for the conopid would have been too short (7 days) if the attack had occurred during the first day of field exposure.

### Statistics

All statistical tests were done in R (R Development Core Team 2021). To test for differences between parasitized and unparasitized bees in the likelihood of staying out at night, we ran a Kruskal test using the total number of times (maximum possible = 4) that each bee stayed out at night as the dependent variable. For the effects of parasitism on the number of foraging trips per day and the duration of each foraging trip, we built statistical models with individual bee as a repeated measure, foraging trips per day and foraging trip duration as dependent variables, and day of experiment and parasitism status (and their interaction) as factors using the package lme4 (Bates et al. 2015). To test the effect of parasitism on resources returned to the colony per foraging trip, we ran a similar test using proportional body weight increase between leaving and entering the colony as the dependent variable but also included whether the trip was completed after spending the night out of the colony as a factor in the model. Only trips with positive resource return (93% of trips) were included in this estimate to reduce the effect of defecation trips and colony cleaning trips on the resource return estimate. To test whether colony departure time for the first trip of the day was different between parasitized and unparasitized bees, we ran a similar model as the others using departure time of first trip as the dependent variable and also included whether the trip followed an overnight trip. In all repeated measures analyses, if the model residuals did not appear normal, we transformed the dependent variable. Since the parasite grows substantially each day in the bee gut, we expected that any effects on bee behavior would increase over time and thus the interaction in the statistical models would be most sensitive to detecting the effects of the parasite on response variables. Experiment day was considered an ordered categorical variable in the statistical models but nearly all results were qualitatively identical if day was considered a numerical value. Significance was assessed using the package car (Fox and Weisberg 2019) through analysis of variance using type III sum of squares. If we found a significant interaction, then we examined the relationship with an interaction plot to show where the apparent effect of the growing parasite became detectible, which was just after day 2 for both trips per day and resources per trip, and just after day 1 for trip duration. We then compared the daily rates for all three variables from the day following the statistical interaction until the end of the experiment for differences between parasitized and unparasitized bees. To look at the cumulative effects of the parasite on total resources returned from the onset of the interaction until the end of the experiment, we estimated the total resources collected per bee between days 3 and 5. We did not have resource estimates for all trips taken during this time period because of a failure of the scale to stabilize in some cases. To account for this problem, we calculated a resource return rate per trip per day for each bee based on the trips that were quantified, and multiplied that estimate by the actual number of trips taken that day. We tested the significance of parasitism on total resources returned (standardized by exit body weight) with a one-way analysis of variance, with cumulative resources returned per bee as the dependent variable and parasitism status as the factor.

## Results

Of the 30 bees exposed to conopids in the lab, 21 bees were observed to be attacked 1–4 times in the lab. The bee attacked 4 times died shortly after the last attack, but the others quickly resumed flying activity in the flight cage and appeared healthy upon reintroduction to the colony. Following 5 days of foraging, 34 of the 60 tagged bees were retrieved from the colony, while the others were presumed to have died in the field while foraging. Only 12 of the 30 lab-exposed bees were recovered, whereas 22 of the 30 lab unexposed bees were recovered from the field. Of the 12 lab-exposed bees that were recovered, 6 of them died of a conopid infection 10–12 days after lab exposure and were considered to have been infected in the lab. No other bees exposed in the lab died of a conopid infection after foraging. Of the 22 bees that were recovered from the unexposed lab group, 5 died of conopid infections picked up in the field, 12–18 days after being allowed to forage. These 5 bees were excluded from subsequent analyses comparing infected and uninfected bees given that they would have younger infections in various stages of development during field observations. Finally, the dataset was further reduced by limiting analyses only to bees that foraged for all 5 days of the field experiment. This was done to guarantee similarity of field conditions among foragers (weather, floral availability, etc.) on all the days in which they were being compared. This produced a core dataset for 21 bees, 6 that had been lab infected and 15 that had not been lab infected and did not acquire an infection in the field.

A total of 718 foraging trips were recorded over the 5-day period for these 21 bees and formed the dataset for trip frequency. Of these, 402 trips were able to be fully scored for bee exit and enter weights and formed the core dataset for resource return. The primary loss of scale data was due to a failure of the scale to stabilize, which occurred when several bees crossed the scale at once or when debris bridged the gap between the exit tube and the scale balance mechanism and had to be removed.

**Fig. 3:**
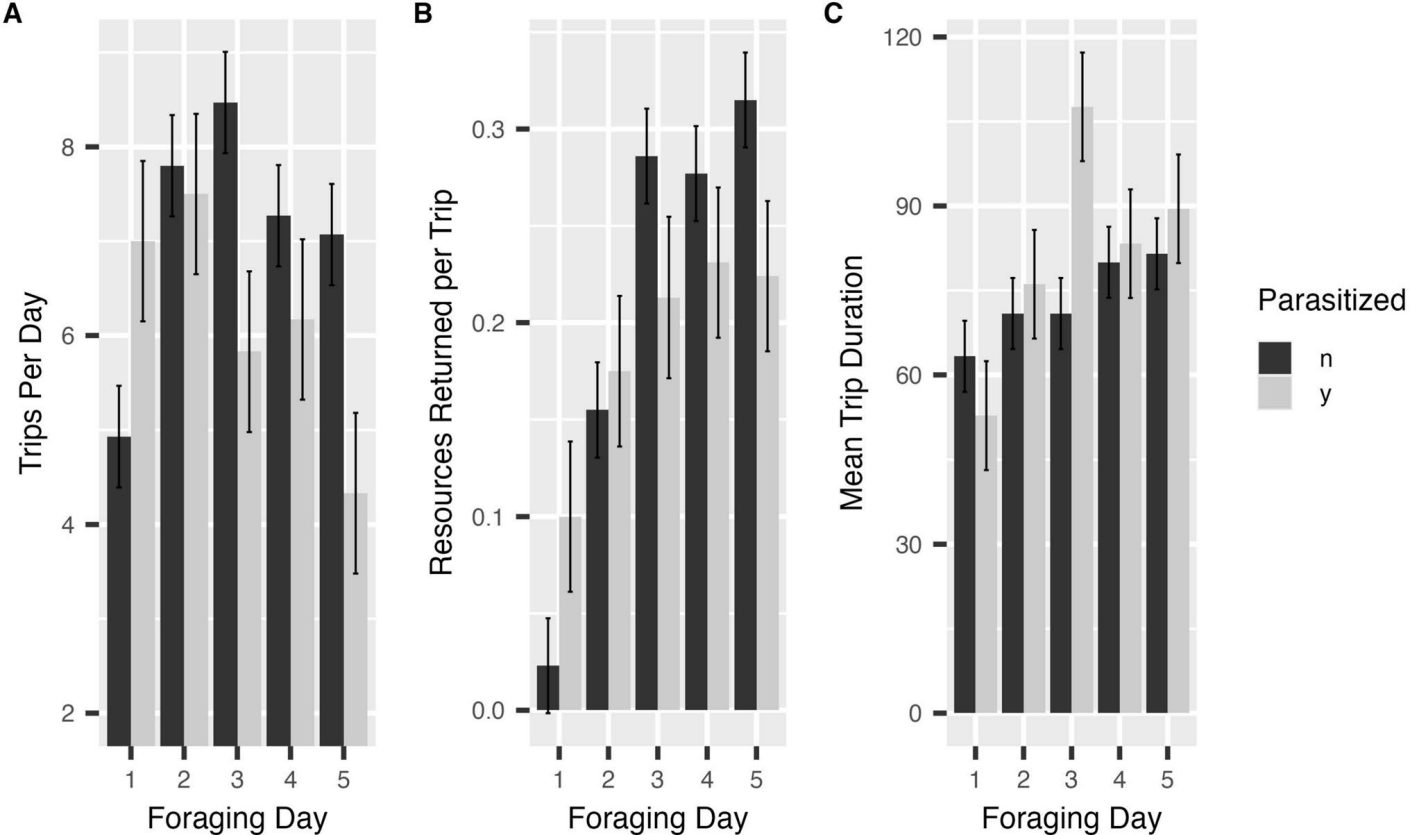
Change in foraging parameters per day for parasitized and unparasitized bees (mean ± SD). **A** Number of foraging trips per day. **B** Amount of resources returned to the colony by individual bees on each trip. Resource return measured as the proportional increase in body weight of a returning forager compared to its weight as a departing forager. **C** Foraging trip duration (minutes) by bumblebee workers

**Fig. 4:**
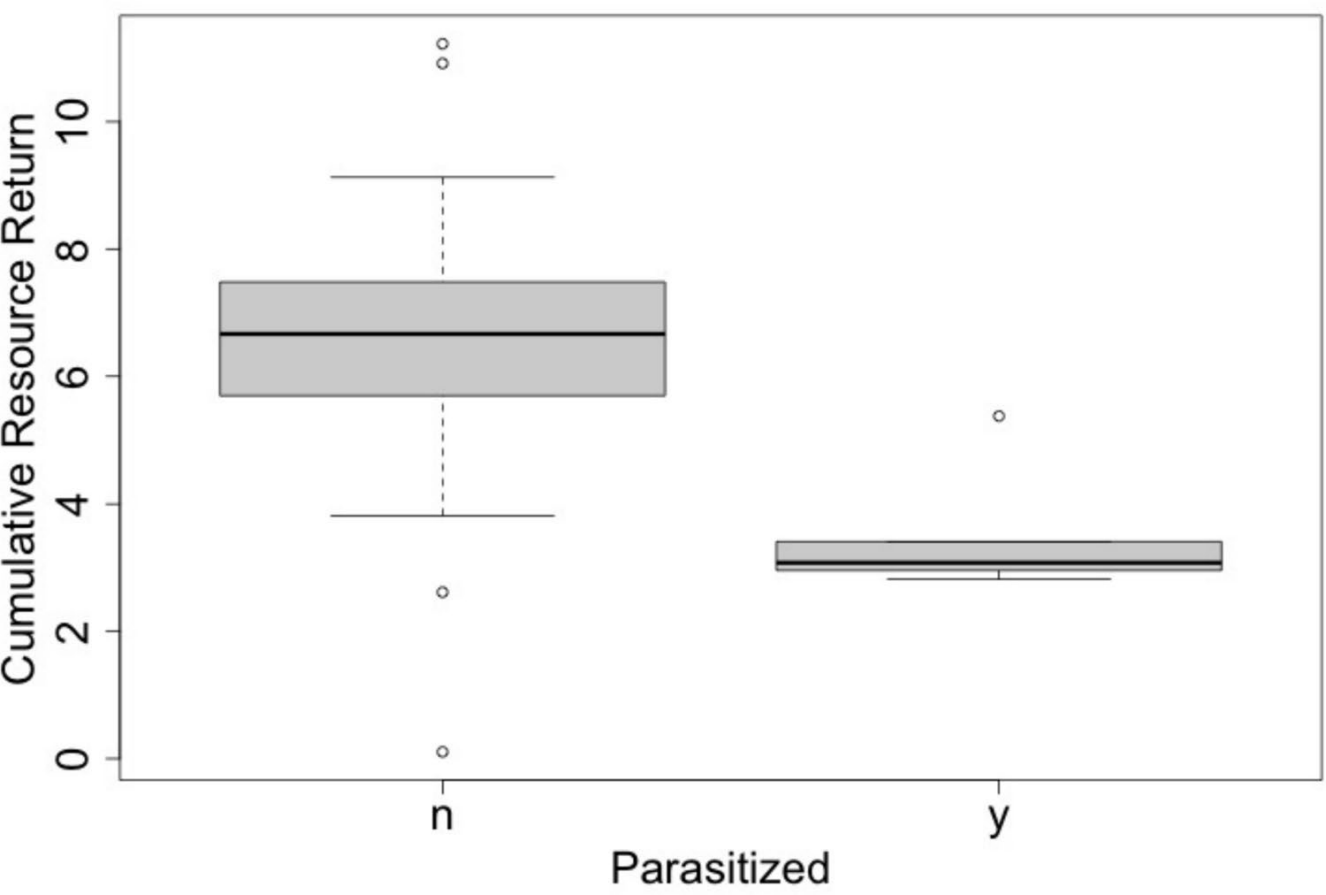
Cumulative resources returned over the last 3 days of the experiment. Resources standardized for body size as the proportional increase in return body mass over body mass during exit

**Fig. 5:**
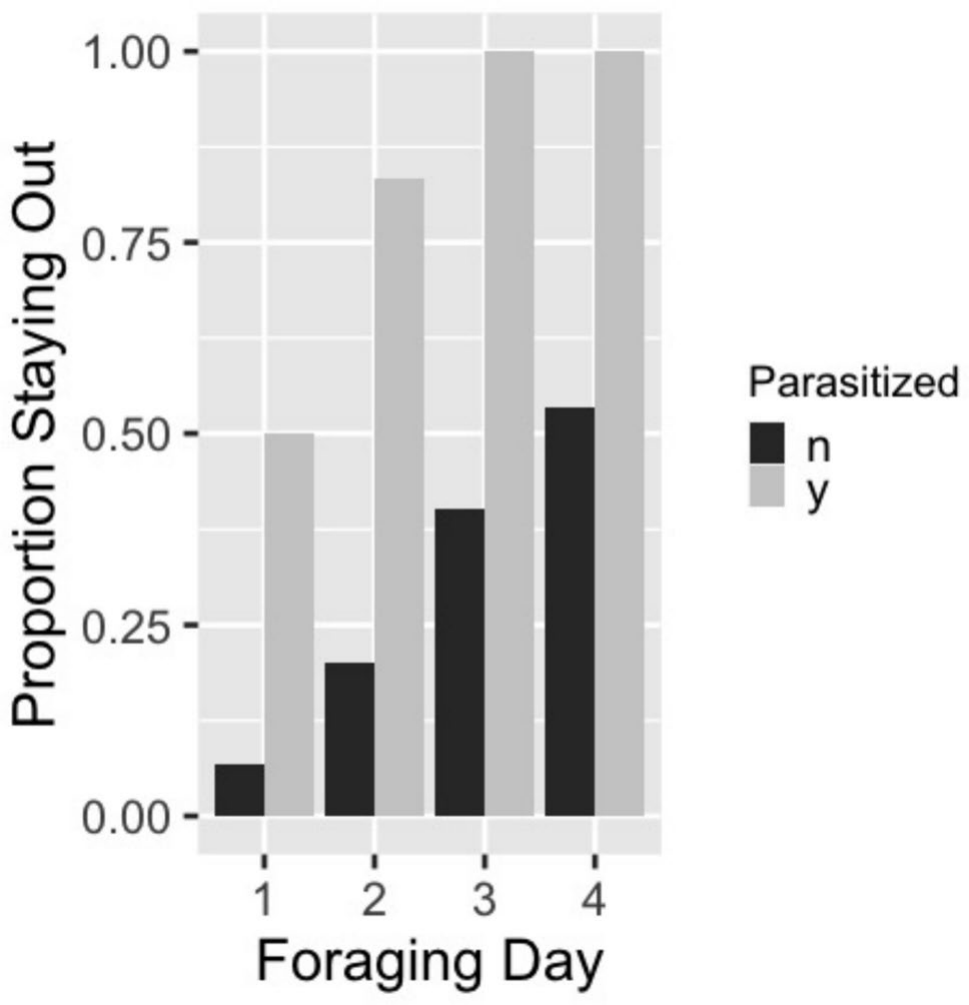
Proportion of bees staying out of the colony at night after each foraging day

The total number of trips taken ranged from 29 to 49 trips per bee. Over the 5-day period, unparasitized bees took a total of 35.2 ± 2.0 (mean ± SE) trips compared to 30.8 ± 3.16 trips for parasitized bees (*F* = 1.577, *p* = 0.224, 1-way ANOVA). Trips per bee per day varied significantly across the days of the experiment and there was a significant interaction between foraging day and parasitism status (Table 1A), with parasitized bees appearing to take fewer trips from days 3 to 5 (Fig. 3A). An interaction plot showed the change in effect starting on day 3. Looking at foraging trips on just those last 3 days, parasitized bees took significantly fewer trips per day (*p* = 0.009). The amount of resources bees returned to the colony, measured as a proportional increase in their body weight, also varied across foraging days and with an interaction of foraging day and parasitism status (Table 1B) (Fig. 3B). An interaction plot showed this shift in effect starting at day 3. Although this pattern was in a similar trend to that of trips per day, parasitized bees did not carry significantly fewer resources per trip than unparasitized bees over these last 3 days (*p* = 0.087). Combining the number of trips per day with the quantity of resources per trip, parasitized bees returned ~ 50% fewer total resources than unparasitized bees over the last 3 days of the experiment (*p* = 0.026, Fig. 4). The amount of food returned to the colony was not influenced by whether the bee stayed out overnight before the trip. Duration of foraging trips per bee varied over time and with an interaction between time and parasitism status (Table 1C). An interaction plot showed a shift in effects starting on day 2. Once again, the general pattern of mean values for trip duration was similar to other tests, with parasitized bees on average taking more time than unparasitized bees toward the end of the experiment (Fig. 3C). The trip duration per day for parasitized bees from day 2 to 5 was only marginally longer than for unparasitized bees (*p* = 0.054). Bees infected by conopid flies spent more nights away from the colony than uninfected bees (median of 3.5 versus 1 for infected versus uninfected bees, Wilcoxon rank sum test with continuity correction, *W* = 8, *p* value = 0.003). Half the infected bees stayed out of the colony the first night and all stayed out by the last night. Only one of the 15 unparasitized bees stayed out of the colony the first night, but over half did so by day 4 (Fig. 5). Bees that spent the night outside the colony left the colony the next day for their first trip about an hour later than bees that spent the night inside the colony (Table 1D) but this was not influenced by parasitism status.

**Table 1:** Comparison of parasitized and unparasitized bees across four metrics of foraging over time

	*F*	Df	*P*
A) Trips per day			
(Intercept)	314.453	1	2.803e-13***
Parasitized	1.577	1	0.224
Day	4.660	4	0.002**
Parasitized*day	6.911	4	8.452e-05***
B) Resources returned			
(Intercept)	117.039	1	5.6e-13***
Parasitized	0.001	1	0.977
Day	41.079	4	< 2.2e-16***
Overnight	0.099	1	0.754
Parasitized*day	4.761	4	< 0.001***
Parasitized*overnight	0.605	1	0.437
C) Trip duration			
(Intercept)	6859.846	1	< 2.2e-16***
Parasitized	1.023	1	0.325
Day	12.419	4	9.831e-08***
Parasitized*day	3.719	4	0.008**
D) Time departing the colony for first trip each day			
(Intercept)	2706.773	1	< 2.2e-16***
Parasitized	0.0116	1	0.916
Day	5.9760	3	0.001**
After Overnight	15.8635	1	< 0.001***
Parasitized*day	4.3436	3	0.008**
Parasitized*after Overnight	0.0362	1	0.849

*p<0.05, **p<0.01, ***p<0.001

## Discussion

Infected bees showed diminished foraging performance by the third day of the experiment. The primary effect was taking fewer trips per day, but there were also non-significant trends toward carrying less food per trip, and taking more time per trip. This pattern continued to the end of the experiment at foraging day 5, which represented either the 8th or 9th day since they were infected in the lab. These represented advanced infections given that the bees died of their infections 2–3 days after being returned to the lab with their entire abdominal cavity occupied by the parasite. Previous work (Malfi and Roulston 2014) found that foraging bumblebees are sometimes caught with late stage conopid infections that were within hours of killing the bee, and thus the bees often work for the colony until close to the end of their life. But here we show their escalating deduction in foraging efficiency as the parasite develops, an effect that probably extends the additional 2–3 days to bee death. Prior work has shown that bumblebees parasitized with a conopid choose different host plants to visit (Schmid-Hempel and Schmid-Hempel 1990; Schmid-Hempel and Stauffer 1998) and are less likely to carry pollen than unparasitized bumblebees (Schmid-Hempel and Schmid-Hempel 1991). Thus, these pre-lethal effects on bumblebee foraging magnify any colony-level effects attributable to the shorter lifespans of parasitized bumblebee workers (Müller and Schmid-Hempel 1992; Gillespie 2010; Malfi et al. 2018).

Despite the significant change in parasitized bee behavior by foraging day 3 (day 6–7 following infection), it is notable that foraging efficiency was not reduced earlier. The change in foraging efficiency is likely attributable to an increasing drain on bee physiological resources as the parasite grows. For the European conopid species *Physocephala rufipes*, the egg stage lasts about 2 days (Schmid-Hempel and Schmid-Hempel 1996), a time in which the main impact on the host would likely be any trauma directly related to oviposition. During the first two larval stages, occurring over the next ~ 7 days, the larva remains relatively small and feeds primarily on hemolymph (Abdalla et al. 2014). During the 3rd larval instar, however, the fly larva feeds on internal organ tissue, eventually killing the bee prior to pupating (Schmid-Hempel and Schmid-Hempel 1996). It is not surprising that the bee becomes a poorer forager at a time in which its internal organs are being consumed by a parasitoid. It is, perhaps, more surprising that it forages as reliably as it does just prior to death.

Our data confirm the tendency of bees infected by conopids to stay out of the colony at night as described in Europe (Müller and Schmid-Hempel 1993). In their experiments, Müller & Schmid-Hempel (1993) dissected foragers found in the colony at night and those returning to the colony after a night out and found that the returning ones were much more likely to be parasitized. In a lab experiment, they also showed that parasitized workers lived longer and suppressed parasitoid growth more if subjected to a constant cool lab temperature (19 °C), reflecting outdoor night temperature, than in constant warm temperatures (28 °C), reflecting the temperature of a bumblebee nest. This supports their hypothesis that overnighting behavior is an adaptive mechanism to prolong worker longevity by suppressing a parasitoid infection. In their dataset, bee age was a minor contributor to overnighting behavior. In our dataset, over half of infected bees stayed out by day 2 and over half of uninfected bees stayed out by day 4. This shows that bees initiate overnighting behavior quickly, and that there is a strong effect of experience on overnighting behavior independent of conopid infection. In our dataset, we did not dissect bees to look for immature parasites and cannot say definitively that bees staying out that did not die of conopid infections did not actually have small, failed infections. Prior work with *Bombus impatiens*, however, that did include dissections, found that conopid infections typically succeed in the lab with this species (Davis et al. 2015) and that uninfected workers as scored by dissection often spent nights in the field (unpublished data from Malfi et al. 2018). Thus, there is likely to be at least one as yet undiscovered strong driver of overnighting behavior in addition to conopids, such as other parasites, experience, age, or colony conditions.

While parasitizing bees in the lab proved effective, and was helpful for examining insect behavior relative to a known time of infection, it was also labor intensive for generating replication. The main challenge was collecting sufficient numbers of conopids in the field in a short period of time and having them attack bees in the lab. Despite conopid infections of bumblebees being common in the study region at the time of the study, we seldom readily find many adult female conopids in the field. Lab rearing of conopids could potentially make additional studies easier to carry out. One setback we did not expect was the relatively low return of lab infected foragers at the end of the study. Only 40% of our lab-exposed bees were captured at the end of the experiment, with the missing presumably dying in the field during foraging. In contrast, 74% of non-lab-exposed bees survived over those 5 days. Given that the conopid does not pupate during the time that was available, such loss could be due to trauma of the original attack or to behavioral changes in the field caused by the growing conopid. Neither of these causes is known for conopid effects on bees and could represent additional stresses on bumblebee colony productivity. If mortality due to the physical damage from a conopid attack (or from a subsequent microbial infection) is frequent in the wild, then it would likely represent an even larger effect on bumblebee colonies than the effects recorded here. We hesitate to estimate that effect from our data because we often had multiple attacks on the same bee in short succession, something that may be less likely to occur in the wild. In our trials, we noted that the bee that was attacked the most (4 times) died in the lab shortly after. Bumblebee workers are sometimes found with multiple conopid larvae in the field (Schmid-Hempel and Schmid-Hempel 1996), so we do know that multiple attacks occur, but we do not know how they impact host health independent of the larval parasite. It is likely that this kind of estimate can only be made with additional lab studies.

Overall, we show that measurable reductions on bumblebee productivity are evident about halfway through the period of conopid infection, which may magnify their negative impacts on colony productivity. While infected bumblebees did remain outside the colony shortly after infection, as they do in Europe (Müller and Schmid-Hempel 1993), it was not evident that doing so reduced the trajectory of the infection. Infected bees still died in the 10–12-day period estimated for other species in this system (Schmid-Hempel and Schmid-Hempel 1996). Prior work has shown that bumblebee species differ in their physiological defenses against conopids (Davis et al. 2015). The use of behavioral defenses, such as the use of nighttime cold to suppress an infection, could also vary between species of host and species of parasitoid. The potential use of lab-based infections in this study system, as demonstrated here for the first time, allows future manipulative work to examine host–parasitoid interactions in great detail.

## Data Availability

The datasets used and/or analyzed during the current study are available from the corresponding author on reasonable request and will be published in Figshare upon publication.

## Code availability

All code is available upon request from the corresponding author.
